# Cannabidiol modulates expression of type I IFN response genes and HIV infection in macrophages

**DOI:** 10.3389/fimmu.2022.926696

**Published:** 2022-09-29

**Authors:** Shallu Tomer, Wenli Mu, Gajendra Suryawanshi, Hwee Ng, Li Wang, Wally Wennerberg, Valerie Rezek, Heather Martin, Irvin Chen, Scott Kitchen, Anjie Zhen

**Affiliations:** ^1^ Division of Hematology/Oncology, Department of Medicine, David Geffen School of Medicine at University of California, Los Angeles (UCLA), Los Angeles, CA, United States; ^2^ UCLA AIDS Institute and the Eli and Edythe Broad Center of Regenerative Medicine and Stem Cell Research, David Geffen School of Medicine at University of California, Los Angeles (UCLA), Los Angeles, CA, United States; ^3^ Department of Microbiology, Immunology, and Molecular Genetics, David Geffen School of Medicine at University of California, Los Angeles (UCLA), Los Angeles, CA, United States

**Keywords:** HIV - human immunodeficiency virus, CBD - cannabidiol, ISG (interferon stimulated genes), type I interferons, macrophage

## Abstract

Cannabis (*Cannabis sativa*) is a widely used drug in the United States and the frequency of cannabis use is particularly high among people living with HIV (PLWH). One key component of cannabis, the non-psychotropic (−)-cannabidiol (CBD) exerts a wide variety of biological actions, including anticonvulsive, analgesic, and anti-inflammatory effects. However, the exact mechanism of action through which CBD affects the immune cell signaling remains poorly understood. Here we report that CBD modulates type I interferon responses in human macrophages. Transcriptomics analysis shows that CBD treatment significantly attenuates cGAS-STING-mediated activation of type I Interferon response genes (ISGs) in monocytic THP-1 cells. We further showed that CBD treatment effectively attenuates 2’3-cGAMP stimulation of ISGs in both THP-1 cells and primary human macrophages. Interestingly, CBD significantly upregulates expression of autophagy receptor p62/SQSTM1. p62 is critical for autophagy-mediated degradation of stimulated STING. We observed that CBD treated THP-1 cells have elevated autophagy activity. Upon 2’3’-cGAMP stimulation, CBD treated cells have rapid downregulation of phosphorylated-STING, leading to attenuated expression of ISGs. The CBD attenuation of ISGs is reduced in autophagy deficient THP-1 cells, suggesting that the effects of CBD on ISGs is partially mediated by autophagy induction. Lastly, CBD decreases ISGs expression upon HIV infection in THP-1 cells and human primary macrophages, leading to increased HIV RNA expression 24 hours after infection. However, long term culture with CBD in infected primary macrophages reduced HIV viral spread, suggesting potential dichotomous roles of CBD in HIV replication. Our study highlights the immune modulatory effects of CBD and the needs for additional studies on its effect on viral infection and inflammation.

## Introduction

Cannabis (*Cannabis sativa*) is a widely used drug in the United States and the frequency of cannabis use is particularly high among people living with HIV (PLWH) (1). The two major active components of the cannabis sativa plant, the psychotropic-trans-Δ9-tetrahydrocannabinol (THC) and the non-psychotropic-cannabidiol (CBD) exert a wide-variety of biological actions including anticonvulsive, analgesic, and anti-inflammatory effects (2). Heavy cannabis usage was associated with reduction in frequency of activated immune cells in PLWH (3) and lower plasma HIV RNA among recently infected drug users (4). Exposure to cannabis is recently reported to be associated with a lower likelihood of neurocognitive impairment in PLWH (5). However, results are still conflicting regarding the health benefits/risk of cannabis usage for PLWH (6–8).

CBD, one of the primary non-psychotropic components in the cannabis plant, is well tolerated and may have potential anti-inflammatory effects (9, 10). A highly purified form of CBD oil (Epidiolex) derived from *C.Sativa* is FDA approved for treatment of rare forms of seizures (11). With increased societal acceptance of recreational cannabis and CBD oil for putative medicinal use in many parts of United States, the exposure to CBD is increasing (12). However, the exact mechanism of action through which CBD interacts with the immune system and immune cell signaling are poorly understood. Intriguingly, a recent report showed that CBD could inhibit SARS-CoV-2 replication in lung epithelial cells, suggesting that CBD may regulate antiviral responses (13).

CBD has been reported to induce autophagy in intestinal epithelium cells and neural cells (14, 15). Autophagy is a homeostatic mechanism involved in the disposal of aggregated proteins and damaged organelles, such as mitochondria (16, 17), as well as eliminating intracellular pathogens (18, 19). It is a conserved cellular process critical for maintaining cellular integrity and metabolism (20). Degradation of proteins by autophagy is critical to maintain cell function during cellular stress, such as nutrient deprivation caused by pathogen replication (21, 22). In addition to maintaining cellular integrity, autophagy also plays critical role in regulating immune functions (23–27). Innate immune responses can activate autophagy (28) and autophagy regulates innate immune responses by modulating the secretion of immune mediators and removing endogenous inflammasome agonists (23, 26, 29). Importantly, autophagy regulates type I Interferons (30, 31), which are central components of the anti-viral immune responses (32, 33).

In this study we examine the effect of CBD on type I interferon responses in human macrophages and its impacts on HIV infection and viral spread in human macrophages.

## Results

### CBD down regulates interferon stimulated genes in stimulated monocytic THP-1 cells and primary macrophages

Type I interferons (IFNs) are key innate and adaptive immune regulators and essential for the development of anti-viral immunity (34). Type I IFNs induces expression of Interferon Stimulated Genes (ISGs) which exert numerous antiviral effector functions (35). Viruses manipulate ISGs system to allow and promote viral infection (36). To study the effect of CBD on Type I IFN responses and other genes, we stimulated THP-1 cells with 2’3’-cGAMP, an endogenous agonist of STING protein which activates cGAS-STING innate DNA sensing pathway and type I IFNs signaling (37). THP-1 cells were treated with vehicle or 10ug/ml CBD and co-stimulated with 2’3’-cGAMP for 8 hours. Afterwards, we extracted RNA for transcriptomics analysis through RNA-seq. We performed differential expression analysis to identify up or down regulated genes in each stimulus condition (**Supplementary Figure 1A**) and the protein-protein interaction network (**Supplementary Figure 1B**).

**Figure 1:**
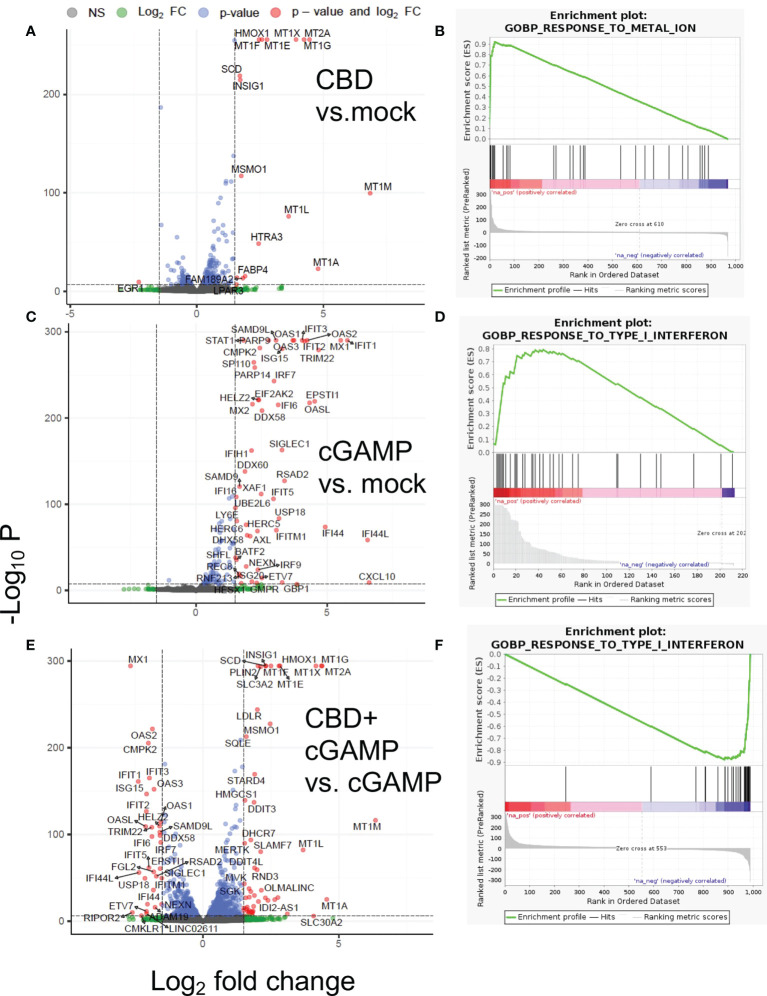
Differential gene expression and gene set analysis of RNA-seq data. THP-1 cells were treated with vehicle or 10ug/ml CBD and co-stimulated with 2’3’-cGAMP for 8 hours. Afterwards, we extracted RNA for transcriptomics analysis through RNA-seq. Volcano plot and gene set analysis comparing CBD treatment alone to mock **(A, B)**, 2’3-cGAMP treatment alone to mock **(C, D)**, 2’3’-cGAMP and CBD cotreatment compared to 2’3’-cGAMP treatment alone **(E, F)**.

The differential gene expression analysis and gene set enrichment analysis (GSEA) of RNA-seq data revealed that in THP-1 cells that were treated by CBD alone (**Figure 1A**) showed significant upregulation of Metallothionein (MT) gene family (MT2A, MT1G, MT1X, MT1F, MT1E, MT1M, MT1L) and heme oxygenase 1 (HMOX1), which are responsible for metal homeostasis and oxidative stress response (**Figure 1B**) (38). Lipid related genes as well as heat shock protein family genes were also upregulated by CBD (**Supplementary Data 1A**). Expectedly, stimulation by 2’3’-cGAMP lead to induction of Interferon Stimulated Genes (ISGs) such as MX1, OAS1, IRF7, ISG15, IFIT1, IFIT6, CXCL10 etc., that are critical for mounting anti-viral responses (**Figures 1C, D**) (39). Interestingly, compared to 2’3’-cGAMP stimulation alone, co-stimulation with both CBD and 2’3’-cGAMP led to dampened expression of ISG, indicating significant downregulation of ISGs in presence of CBD (**Figure 1E**). Attenuation of ISGs stimulation of 2’3’-cGAMP by CBD is also indicated by GSEA (**Figure 1F**). The data discussed in this publication have been deposited in NCBI’s Gene Expression Omnibus and are accessible through GEO series accession number GSE201508 (https://www.ncbi.nlm.nih.gov/geo/query/acc.cgi?acc=GSE201508).

**Figure 2:**
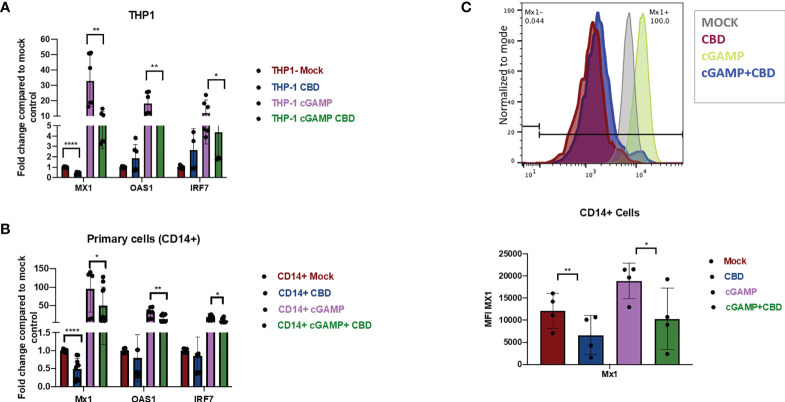
CBD down regulates the expression of ISGs in monocytic cell line THP-1 and primary macrophages. **(A)** THP-1 cell lines were treated with vehicle or CBD overnight and stimulated with or without 2’3’-cGAMP for 8hrs. Expression levels of ISGs MX1, OAS1, IRF7 was measured by real time PCR. **(B)** CD14+ cells were sorted from healthy PBMCs followed by differentiation with M-CSF for 5 days. Afterwards, cells were treated with CBD for 8hrs. Expression levels of MX1, OAS1, IRF7 was measured by real time PCR. **(C)** ISG Mx1 protein in CD14+ primary macrophages were measured by flow-cytometry. *p<0.05, **p<0.005, ****p<0.0001.

To cross-validate the effect of CBD on Type I IFN responses in THP-1 cells, THP-1 cells were treated with vehicle or 10ug/ml CBD and stimulated with 2’3’-cGAMP for 8 hours. Afterwards, expression of Interferon response gene MX1, OAS1, IRF7, ISG15, IFIT1 (**Figure 2A** and **Supplementary Figure 2A**) were measured by real time PCR as described previously (40, 41). Treatment of CBD alone did not significantly alter the expression level of many ISGs in unstimulated THP-1 cells. In contrast, CBD cotreatment of cGAMP-stimulated THP-1 cells significantly attenuated all measured ISGs (**Figure 2A** and **Supplementary Figure 2A**). Additionally, we cross validated our RNAseq data that CBD induces MT2A and HMOX1 expression in THP-1 and primary macrophages, regardless of 2’3’-cGAMP stimulation (**Supplementary Figures 2C, D**).

To examine if CBD has similar effects in human primary macrophages, CD14+ monocytes were sorted from PBMCs of healthy donors and differentiated into macrophages by culturing with M-CSF as previously described (42). Cells were treated with vehicle or CBD and then stimulated with 2’3’-cGAMP for 8 hours. Similar to THP-1 cells, we observed significant attenuation of ISGs expression in 2’3’-cGAMP stimulated macrophages by CBD treatment (**Figure 2B** and **Supplementary Figure 2A**). We further confirmed down regulation of ISG MX1 protein by CBD treatment in 2’3’-cGAMP stimulated macrophages with flow cytometry (**Figure 2C**), suggesting that CBD could down regulate key ISG expression in treated cells.

### CBD induces autophagy to attenuate cGAS-STING signaling

**Figure 3:**
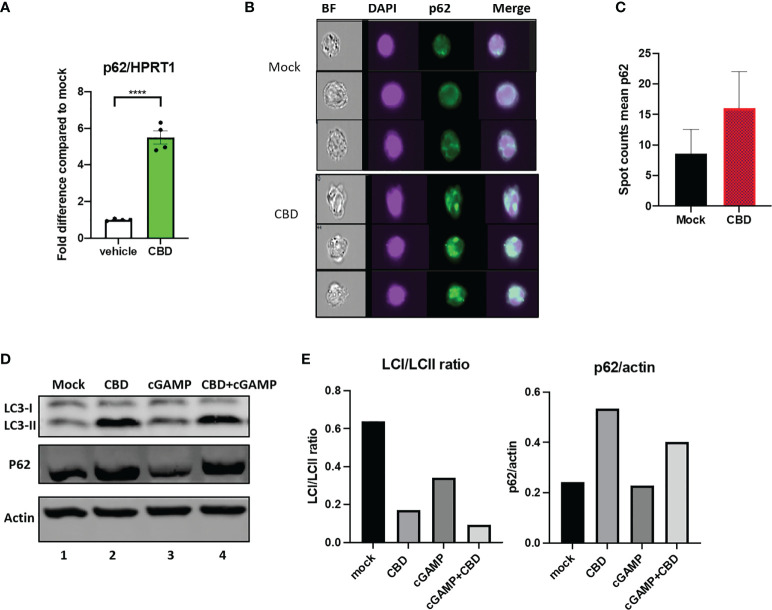
Cannabidiol treatment leads to increased autophagy activity. **(A)** Real time PCR analysis of p62 expression in THP-1 cells treated with either vehicle or CBD for 8 hours. **(B)** THP1 cells were treated with CBD overnight, fixed and stained with DAPI, anti-p62 and analyzed by ImageStream. **(C)** Bar graph showing mean Spot count of p62 for mock and CBD treated THP1 analyzed by ImageStream IDEA software. **(D)** Western blot of THP1 cells treated with DMSO, CBD, cGAMP or CBD+cGAMP overnight. **(E)** Bar graph showing LCI/LCII ratio and p62/actin of the western blot analyzed by Image Lab Software. ****p<0.0001.

In our RNAseq analysis, we observed that CBD upregulate autophagy receptor SQSTM1/p62 expression regardless of 2’3’-cGAMP treatment (**Supplementary Figure 1A**). SQSTM1/p62 appears to be associated with both MT gene family, heat shock ISGs and heat shock gene protein family genes by STRING protein-protein network analysis (**Supplementary Figure 1B**). We further cross validated this finding by real time PCR analysis of THP-1 cells treated with either vehicle or CBD (**Figure 3A**). The autophagy receptor p62 plays an important role in delivering ubiquitinated cargoes for autophagic degradation (30). Importantly, p62 mediates attenuation of cGAS-STING type I IFN response by directing STING for autophagy mediated degradation (30).

To examine if CBD treatment alone can increase formation of autophagosome, THP1 cells were treated with vehicle or 10ug/ml overnight. Afterwards, cells were stained with p62 and imaged by ImageStream (43). As shown in **Figures 3B, C**, we examined increased number of p62 containing puncta (in green) by ImageStream in CBD treated conditions (16±6) as compared to vehicle alone (8.5±4), suggesting elevated autophagy. We then performed western blotting using THP1 cells that were treated with vehicle, 10ug/ml CBD alone, 10ug/ml 2’3’-cGAMP alone or CBD plus 2’3’-cGAMP overnight. As shown in **Figures 3D, E**, 2’3-cGAMP treatment alone increases LCII expression but does not significantly alter p62 expression in THP1 cells. In contrast, we observed increased p62 and LC3II expression in cells treated with CBD and 2’3’-cGAMP plus CBD, resulting in lower LCI/LCII ratio in CBD treated samples, suggesting increased autophagy activity.

**Figure 4:**
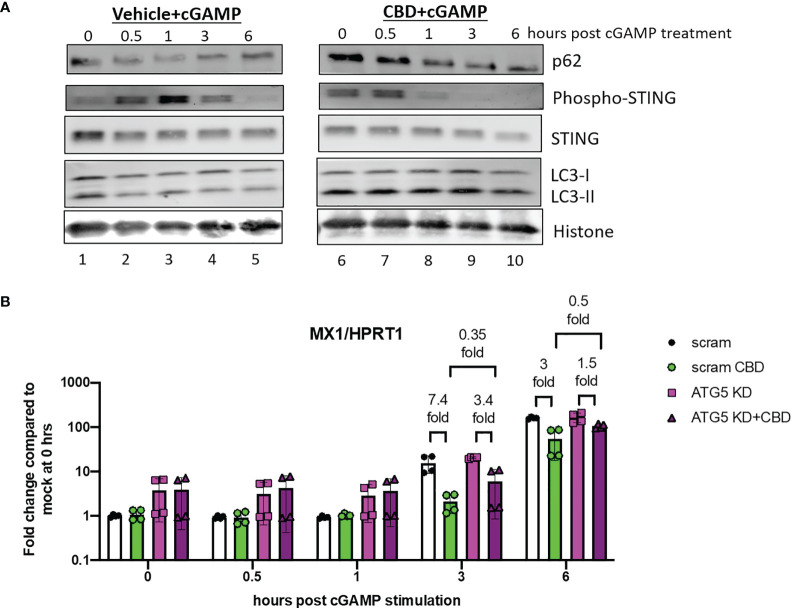
CBD leads to rapid down regulation of phosphor-STING expression after 2’3’-cGAMP stimulation. **(A)** Western blot analysis of THP-1 cells that were treated with either vehicle or CBD, followed by 2’3-cGAMP stimulation. Cells were harvested at 0, 0.5, 1, 3, 6 hours post stimulation. The expression of autophagy markers (p62/LC3-II), STING and phospho-STING, and histone protein control in vehicle (lane 1-5) and CBD treated (lane 6-10) cells were analyzed. **(B)** THP-1 cells were transduced with either CRISPR/Cas9 system with either scramble or ATG5 targeting guide RNA. Afterwards, cells were treated with either vehicle or CBD, followed by 2’3’-cGAMP stimulation. Cells were harvested at 0, 0.5, 1, 3, 6 hours post stimulation and MX1/HPRT1 expression was measured by real time PCR.

To examine the effects of CBD on autophagy and regulation of cGAS-STING signaling, we treated THP-1 cells with either vehicle or CBD overnight, followed by 2’3’-cGAMP stimulation and harvested cells 0, 0.5, 1, 3, 6 hours after 2’3’-cGAMP stimulation. Prior to 2’3’-cGAMP treatment, we observed significant increased expression of p62 and lipidated LC3-II (**Figure 4A**, comparing lane 6 to lane 1 at 0 hour) in CBD treated cells compared to control, indicating increased autophagy activity by CBD treatment. In vehicle treated cells after 2’3’-cGAMP stimulation, we observed strong upregulation phospho-STING 0.5 to 3 hours post stimulation in control cells (**Figure 4A** lane 1-3), followed by down regulation of both STING and phospho-STING, as reported previously (30). In contrast, in CBD treated cells, upregulation of phospho-STING by 2’3’-cGAMP was subdued, and phospho-STING was rapidly down regulated within 1 hours of stimulation (**Figure 4A** lane 6-8). These results indicate that CBD induces autophagy in THP-1 cells and down regulates STING and phospho-STING level after 2’3’-cGAMP stimulation.

To examine if autophagy is important for CBD attenuation of ISGs expression, we transduced THP-1 cells with lenti-X CRISPR/Cas9 systems that express CRISPR/Cas9 and either scrambled guide RNA or guide RNA against autophagy protein ATG5. ATG5 knockdown (ATG5 KD) cells has impaired autophagy activity as we have reported previously (44). Scramble or ATG5 KD cells were treated with CBD overnight and stimulated with 2’3’-cGAMP. Cells were harvested at 0, 0.5, 1, 3, and 6 hours post stimulation. As shown in **Figure 4B**, we observed early elevation of ISG MX1 expression in scramble vehicle treated cells as early as 3 hours post stimulation as measured by real time PCR. CBD treatment in scramble THP-1 cells lead to early attenuation of ISG MX1 expression 3 hours after stimulation. Interestingly, we observed reduced CBD suppression of MX1 in ATG5 KD cells at 3 hours (7.4 fold reduction by CBD in scramble cells and 3.4 fold in ATG5 KD cells) and 6 hours (3 fold reduction by CBD in scramble cells and 1.5 fold in ATG5 KD cells). Mean expression of MX1 is lower in CBD treated scramble cells than CBD treated ATG5KD cells (0.35 fold at 3hours, 0.5 fold at 6 hours after stimulation), suggesting attenuation of ISGs by CBD is partially mediated by autophagy.

### CBD suppresses ISG MX1 expression and affects HIV replication in macrophages

ISGs play important roles in suppressing HIV replication during early infection (45). Many reports have suggested that sensing of HIV-1 entry itself triggers a broad expression of Type-1 IFN response in primary macrophages, conferring an early protection against virus (46). Suppression of these ISGs during initial exposure results in the spread of virus (46).

**Figure 5:**
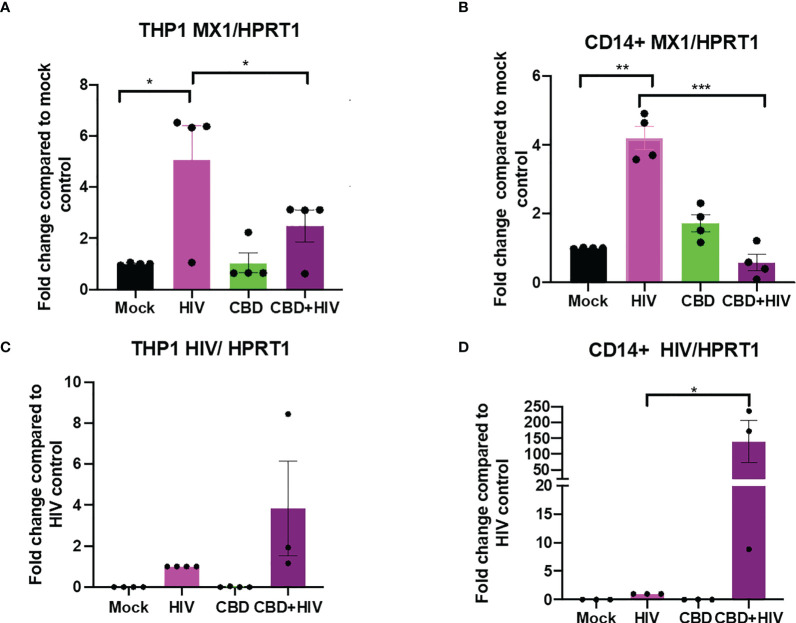
CBD suppresses ISG MX1 expression level and increase HIV RNA level 24 hours after infection in THP-1 cells and macrophages. THP-1 cells were stimulated for 5 days with PMA and primary monocytes were differentiated using M-CSF for 5 days. Cells were treated with vehicle or CBD for 24 hours followed by infection with HIV for 24 hours and harvested. Fold change of expression level of MX1/HPRT1 compared to mock in **(A)** THP-1 cells and **(B)** CD14+ primary macrophages. Fold change of HIV RNA expression level compared to vehicle treated HIV infected **(C)** THP-1 cells or **(D)** CD14+ primary macrophages as measured by real time PCR. *p<0.05, **p<0.005, ***p<0.0005.

To study the effect of CBD in HIV infected cells, we treated either differentiated THP-1 cells or primary macrophages with CBD for 24 hours followed by HIV infection for another 24 hours. We found that in mock treated THP-1 cells and primary macrophages, HIV infection moderately upregulate ISG MX1 expression (**Figures 5A, B**). In contrast, cells that were treated with CBD showed lower level of MX1 expression after HIV infection (**Figures 5A, B** **)**. In addition, we observed that both CBD treated THP-1 cells and primary macrophages have higher level of HIV RNA as compared to control 24 hours after infection (**Figures 5C, D**). This suggests that down regulation of ISGs by CBD may result in increased HIV entry and expression during early infection.

**Figure 6:**
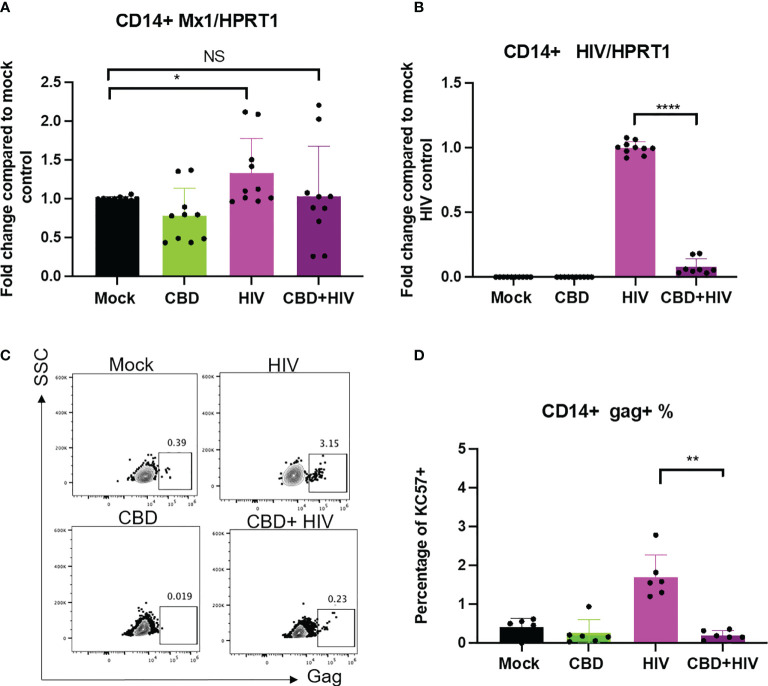
CBD affects HIV viral spread in primary macrophages. CD14+ cells were magnetically sorted and differentiated with M-CSF for 5 days followed by treatment with vehicle/CBD overnight. After treatment, cells were infected with HIV for 24hrs with vehicle/CBD treatment for another 8 days. **(A)** ISG MX1 expression level as measured by real time PCR. **(B)** HIV RNA level as measured by real time PCR. **(C)** Representative flow plot of cells stained with anti-gag antibody after 8 days of infection. **(D)** Summary of gag+% cells. *p<0.05, **p<0.005, ****p<0.0001, NS, non-significant.

To examine how CBD affects HIV infection in long term culture, human primary macrophages were treated with vehicle/CBD overnight, followed by HIV infection in the presence or absence of CBD treatment for 8 days. We observed increased LC3-II, lower LC3-I/II ratio and p62 mRNA expression level in cells infected and treated with CBD for 8 days, suggesting continuous elevation of autophagy (**Supplementary Figure 3**). Similar to previous reports (47), we found that 8 days of HIV infection only leads to low upregulation of ISG MX1 in mock treated macrophages (**Figure 6A**), likely due to HIV counteraction of type I IFN responses (47). In addition, we did not observe significant difference in ISG expression comparing CBD treated to mock treated cells that were infected with HIV (**Figure 6A**). Interestingly, we observed significantly decreased viral spread in CBD treated macrophages as measured by either expression of HIV RNA (**Figure 6B**) or expression of Gag (**Figures 6C, D**). Our data suggests the potential dichotomous roles of CBD in HIV replication.

## Discussion

CBD is one of the main biologically active compounds found in cannabis, which is widely used in US, recreationally and medicinally (48). CBD is non-psychoactive and has been reported to have anti-inflammatory and antioxidant effects (49). Previous research has primarily focused on CBD’s effects for treating epilepsy and seizure (50–52). A recent report suggests that CBD can inhibit SARS-CoV-2 in lung cells by the induction of ER stress and innate immune response (13). However, very little is known about CBD’s effects on immune cells such as macrophages, which are critical innate immune sensors and target of HIV infection.

Here we report that CBD can induce global transcriptional changes in monocytic THP-1 cells. Importantly, we observed that CBD attenuated cGAS-STING activation of interferon stimulated genes in both THP-1 cells and primary human macrophages. Interestingly, we found that CBD can induce autophagy activity in THP-1 cells. Previous reports show that STING directly activates autophagy to finetune type I IFN responses by stimulating autophagy-dependent STING degradation (31). We observed differential STING and phosphor-STING expression level in CBD treated and control cells. To examine if CBD treatment affects gene expression of kinases and phosphatases that regulate STING function, we searched our RNAseq database for differential expression of these genes (53). We observed no significant change of TBK1 and minimum increase in ULK1 expression (1.18 fold) by CBD treatment (54, 55). We also did not observe significant changes in phosphatases related to STING inhibition such as PPP6C (56). In contrast, we observed rapid down regulation of phosphor STING and STING post 2’3’-cGAMP stimulation in CBD treated cells, which may be a result of increased autophagy mediated degradation (31). Additional in-depth studies are needed to investigate how CBD affect STING phosphorylation and degradation.

Autophagy mediated STING degradation is dependent on autophagy receptor SQSTM1/p62 (30). We observed both upregulation of p62 RNA and protein expression as well as increased autophagy activity in CBD treated cells. In addition, CBD attenuation of ISGs is reduced in autophagy deficient cells, suggesting that CBD regulation on ISGs is partially mediated by autophagy. Apart from p62, we also observed that CBD upregulates hypoxia related genes and metallothionein genes regardless of 2’3’-cGAMP stimulation (**Supplementary Figure 1**). p62 links autophagy and Nrf2/Keap1/ARE signaling, which is a redox sensitive signaling axis that functions to protect cells against oxidative stress and environmental toxicants (57). Recent reports suggest that CBD can activate the Nrf2/Keap1/ARE pathway and attenuate production of ROS (58, 59). P62 is also a target gene for Nrf2 transcription factor, which binds to the ARE (Antioxidant response element) in p62 promoter. Increased autophagy and p62 expression also leads to activation of Nrf2 pathway, creating a positive feedback (60) and inducing the transcription of antioxidant genes such as HMOX1, which was observed in our RNAseq study (61). Our study suggests that CBD may exert its anti-inflammatory by inducing autophagy and the p62- Nrf2/Keap1/ARE pathway. Our current RNAseq data set also suggest CBD affects gene expression related to peroxisome proliferator-activated receptors (PPAR) in THP1 cells (such as HELZ2 **Supplementary Figure 1**), which is one of the receptors of CBD (62). However, additional studies are needed to determine if CBD induction of autophagy is dependent on PPAR signaling.

Type I Interferons are critical innate responses against viruses through the induction of ISGs that are antiviral effector molecules (32). HIV infection triggers type I IFN responses which are critical for HIV infection control as many ISGs exhibit anti-HIV activities (45, 46, 63, 64). However, HIV also impairs functions of antiviral ISGs and escapes effective innate recognition (64–66). Our study showed that CBD abated ISG elevation during early HIV infection, and CBD treated cells express higher level of HIV RNA as compared control cells. Interestingly, we found that after 8 days of HIV infection, with or without CBD treatment, no detectable elevation of ISG is observed in infected macrophages as compared to uninfected control, suggesting suppression and escape of ISGs by HIV as reported before (66). CBD treated infected macrophages, in contrast to early HIV infection (24 hours), showed significantly lower viral RNA level and % of gag+ cells, indicating reduced viral spread in CBD treated cells. Our study suggests a potential dichotomous effects of CBD on early HIV infection and viral spread. The contracting effects of CBD may reflect the dichotomous roles of autophagy during viral infection (67). While autophagy can target viral particles for degradation and therefore reduce viral spread, diverse virus also utilizes autophagy for their benefit during early infection, including HIV-1 (68). Therefore, it is possible that CBD, by increasing autophagy activity, reduces ISGs expression, leading to increased HIV early replication in macrophages. However, heightened autophagy activity may have limited viral spread during prolonged infection as previously reported (69, 70).

In sum, our study revealed novel effects of CBD in human macrophages. We found that CBD treatment increases autophagy activity, leading to attenuation of Type I IFN responses in macrophages upon stimulation. CBD also plays dichotomous role during early HIV infection and viral spread in macrophages. Besides initiating the anti-viral response, type I IFN is implicated in immunopathogenesis and dysfunction during chronic HIV infection (41, 71–73). Therefore, reduction of ISGs by CBD could potentially be beneficial for diseases driven by Type I IFN mediated chronic inflammation. Additional studies on whether CBD impact transcriptome and functions of CD4 T cells, which are major HIV target cells, are needed. Lastly, our study highlights the immune modulatory effects of CBD, animal and clinical studies are needed to further understand the effects and mechanisms of CBD on acute and chronic viral infection and inflammation.

## Materials and methods

### Primary cell culture and differentiation

Healthy Human PBMCs were obtained from Dept. of Virology, UCLA AIDS Institute and monocytes were magnetically sorted using CD14 microbeads, human (Miltenyi Biotec, USA) according to manufactures’ protocol. Briefly, PBMCs were incubated with CD14+ microbeads and passed through LS columns placed in magnetic field with multiple rounds of washing using MACS buffer. Positively selected CD14+ cells were differentiated into macrophages in the presence of R10 (RPMI, 10%FBS, 1% P/S) and Macrophage colony-stimulating factor (M-CSF) growth factor at a concentration of 10ng/ml for 5days.

### Generation of ATG5 knockdown THP-1 cell

THP-1 cells were infected with CRISPR/Cas9 all-in-one lentiviral vector (Multiplicity of Infection: 2) in a medium supplemented with polybrene (4ug/ml) overnight. To improve the efficiency, two consecutive rounds of infections were performed. 48hrs prior to transduction, cells were treated with 2.5 mg/mL of puromycin. After 2 weeks of drug selection, polyclonal stable cell line libraries were established. The target sequence was: sg-52:TGATATAGCGTGAAACAAGT. After selection, ATG5 knockdown THP-1 cells were cultured in RPMI medium containing 10% FBS and puromycin at a final concentration of 1 mg/ml.

### CBD and 2’3’-cGAMP treatment

Human leukemia monocytic cell line (THP-1) and primary macrophages were treated with CBD (10ug/ml) for 8hrs with and without 2’3’-cGAMP (1ug/ml) for ISGs expression and RNA seq. For autophagy experiments, THP-1 cells were treated with CBD (10ug/ml) overnight followed by 2’3’-cGAMP (10ug/ml) stimulation. Cells were harvested at different time points (0h, 0.5h 1h, 3h, 6h) and processed for protein expression of autophagy markers. Scramble and ATG5 knockdown (ATG5KD) cells were treated with CBD (10ug/ml) overnight and treated with 2’3’-cGAMP (1ug/ml) followed by harvesting at different time points (0h, 0.5h 1h, 3h, 6h) to check the expression levels of MX1. For short-term (acute) HIV infection studies, a higher dose of CBD (10ug/ml) was used for 24hrs and for long-term (chronic) experiments, a lower dose of CBD (2ug/ml) was used to treat differentiated CD14+ cells for 8days.

### RNA sequencing

RNA Samples were sent for sequencing at UCLA Technology Center for Genomics & Bioinformatics. Briefly, THP-1 cells were collected after treatment with CBD for 8hrs in the presence and absence of 2’3’-cGAMP stimulation. Samples treated with ethanol served as controls. Three replicates of each experiment were carried out. RNA extraction was done using RNeasy kits (Qiagen). Sample QC and integrity (RIN-equivalent values) was performed using Tapestation Analysis software v3.2, Agilent Technologies. Sequencing was carried out using Illumina Hiseq300 platform.

Raw sequence data of different treatment conditions (in triplicate) were pre-processed for quality using Fastqc. Trimmomatic was used for adaptors and quality trimming. After this, reads were aligned onto human genome (hg38) using STAR aligner (74). SAMtools was used to convert SAM files BAM files. Mapped reads were counted across human genes by using tool featureCounts (75) that provided raw counts data by assigning mapped reads to genes. Differential gene expression analysis with the raw read counts data using R package DESeq2 (76). Raw sequence data and processed data have been deposited to GEO. Gene expression data was analyzed using Gene Set Enrichment Analysis (GSEA) software tool (77, 78) Top 20 ranked Gene Set Enrichment Analysis results were shown in **Supplementary Table 1**. For pathway analysis, STRING (Search Tool for the Retrieval of Interacting Genes/Proteins) software was used. Many of the genes that were differentially regulated by CBD were validated by performing quantitative real time reverse transcription polymerase chain reaction (qRT-PCR). Primers were designed using Primer blast (NCBI).

### HIV infection studies

For acute infection studies, differentiated THP-1 cells and magnetically sorted, differentiated macrophages were treated with CBD (10ug/ml) or vehicle (ethanol) for 24hrs followed by infection with HIV (NL4-3_NFNSX_) for 24hrs and harvested to check the expression levels of MX1 and HIV. For long term infections, differentiated macrophages (CD14+) were treated with CBD (2ug/ml) or vehicle (ethanol) for 24hr followed by infection with 100ng of HIV (NL4-3_NFNSX_) for 24hrs. Cells were washed and cultured in fresh media with or without vehicle/CBD for 8days. After 8days, CD14+ cells were harvested using Accutase.

### Real-time PCR

To measure the levels of ISGs (MX1, OAS1, IRF7, ISG15, IFIT1), MTs (MT2A), HMOX1 and HIV RNA with HPRT1 as an internal control, THP-1 cells and primary cells were harvested for RNA extraction and making of cDNA using the High-Capacity cDNA Reverse Transcription Kit (Thermo Fisher Scientific). Real-time PCR was performed using the following primers and probes:

HIV-1 forward primer: 5′-CAATGGCAGCAATTTCACCA-3′;
HIV-1 reverse primer: 5′-GAATGCCAAATTCCTGCTTGA-3′;
HIV-1 probe: 5′-[6-FAM] CCCACCAACAGGCGGCCTTAACTG [Tamra-Q]-3′;
P62 forward primer: CGGCTGATTGAGTCCCTCTC
P62 reverse primer: CGGCTGATTGAGTCCCTCTC
ISG15 forward primer: GCGCAGATCACCCAGAAGAT
ISG15 reverse primer: GTTCGTCGCATTTGTCCACC
IFIT1 forward primer: GACTGTGAGGAAGGATGGGC
IFIT1 reverse primer: CATCCAGGCGATAGGCAGAG
MT2A forward primer: ATGGATCCCAACTGCTCCTG
MT2A reverse primer: AGCAGCAGCTTTTCTTGCAG
HMOX1 forward primer: GTGCCACCAAGTTCAAGCAG
HMOX1 reverse primer: CAGCTCCTGCAACTCCTCAA

Single Tube TaqMan Gene Expression Assays (Thermo Fisher Scientific): human HPRT1 (Hs01003267_m1), MX1 (Hs00895608_m1), IRF7 (Hs01014809_g1), OAS1 (Hs00973635_m1). Relative mRNA expression was calculated by normalizing each gene to HPRT1 mRNA expression.

### Western blotting

To measure expression level of STING, phosphorylated STING, p62 and LC3II level, THP-1 cells were treated with mock or CBD, followed by 2’3’-cGAMP stimulation and cells were harvested at 0, 0.5, 1, 3 and 6 hours post 2’3’-cGAMP stimulation. Cell lysate were run on SDS-PAGE and analyzed by western blot with the following primary antibodies: anti-STING (clone T3-680), anti-phosphoSTING (Ser366) (clone D7C3S and E9A9K), anti-LC3II (rabbit polyclonal, Abcam ab51520), anti-p62 (clone EPR4844), anti-histone H3 (rabbit polyclonal, Abcam ab18521).

### Antibodies and flow cytometry

The following antibodies were used in flow cytometry: CD45 (clone HI30), CD14 (clone RMO52), MX1 (clone EPR19967), HIV-core antigen (clone KC57). LIVE/DEAD™ Fixable Yellow Dead Cell Stain Kit (Invitrogen). Antibody for cell surface markers and intracellular markers were conjugated to BV785, FITC, PE or RD1 in appropriate combination. The cells were acquired using LSRFortessa flow cytometer and FACSDiva software (BD Biosciences). Data were analyzed using FlowJo v10 software.

### Imaging flow cytometry (ImageStream)

To visualize autophagasomes at a single cell level, multispectral imaging flow cytometry was performed. THP1 cells were treated with vehicle (ethanol) and CBD (10ug/ml) overnight. Cells were harvested, washed with FACS buffer and fixed and permeabilized with Cytofix/Cytoperm buffers (BD Biosciences). Fixed cells were stained with Recombinant Alexa Fluor 488 Anti-SQSTM1/p62 antibody (abcam, ab185015) for 30min, washed with 1X perm buffer followed by staining with DAPI for 10mins. Cells were washed and resuspended with 1% Fixation buffer containing PFA. Samples (10,000 events per sample) were acquired using Amnis Imagestream MarkII Imaging Flow Cytometer (Luminex corp, USA). Single color controls were acquired for compensation analysis. IDEAS (EMD, Millipore) software was used for data collection and analysis of p62 puncta. Spot count feature was used to count puncta of singlet cells dual positive for DAPI and p62 (approximately 5000 cells).

### Statistical analysis

The results are presented as Mean ± SD. Statistical analysis was performed using GraphPad Prism 9.0 software (GraphPad Software Inc., San Diego, CA, USA). All experiments with THP-1 cells were carried out 3-5 independent times unless otherwise stated. Experiments with primary macrophages were carried out independently using sorted CD14+ cells from 6 healthy donors. To compare statistical difference between 2 groups, Mann-Whitney U tests were used. P values less than 0.05 by Mann-Whitney was considered significant (*P < 0.05, **P < 0.005, ****P < 0.001).

## Data availability statement

The datasets presented in this study can be found in online repositories. The names of the repository/repositories and accession number(s) can be found below: GEO, accession ID: GSE201508.

## Author contributions

AZ and ST contributed to conception and the design of the study. ST, WM, GS, HN, LW, WW, VR, and HM performed the study. ST, WM, GS, and AZ wrote the manuscript. SK and IC provided advice to data analysis, interpretation and presentation. All authors contributed to manuscript revision, read, and approved the submitted version.

## Funding

This work was funded by NIAID 1R21AI140866 (to AZ), NIDA R01DA-52841 (to AZ), NIAID R2120200174 (PIs: Xie and AZ), NCI 1R01CA239261-01 (to SK), NIH Grants NIH Grants P30AI28697 (the UCLA CFAR Virology Core, Gene and Cell Therapy Core, and Humanized Mouse Core), U19AI149504 (PIs: SK and IC). This work was also supported by the UCLA AIDS Institute, the James B. Pendleton Charitable Trust, and the McCarthy Family Foundation.

## Acknowledgment

We would like to thank Drs. Ziva Cooper, Kim Faull, Steve Shoptaw and other faculties at UCLA Cannabis Research Initiative as well as faculties at UCLA AIDS institute for providing helpful discussions.

## Conflict of interest

The authors declare that the research was conducted in the absence of any commercial or financial relationships that could be construed as a potential conflict of interest.

## Publisher’s note

All claims expressed in this article are solely those of the authors and do not necessarily represent those of their affiliated organizations, or those of the publisher, the editors and the reviewers. Any product that may be evaluated in this article, or claim that may be made by its manufacturer, is not guaranteed or endorsed by the publisher.
