# Supplementary material for: Cannabidiol modulates expression of type I IFN response genes and HIV infection in macrophages
Source: Front Immunol. 2022 Sep 29;13:926696. doi: 10.3389/fimmu.2022.926696 (PMC9560767; doi:10.3389/fimmu.2022.926696)
Supplement: Supplementary file 1 [file DataSheet_1.pdf]

Supplementary Figure and Figure Legends

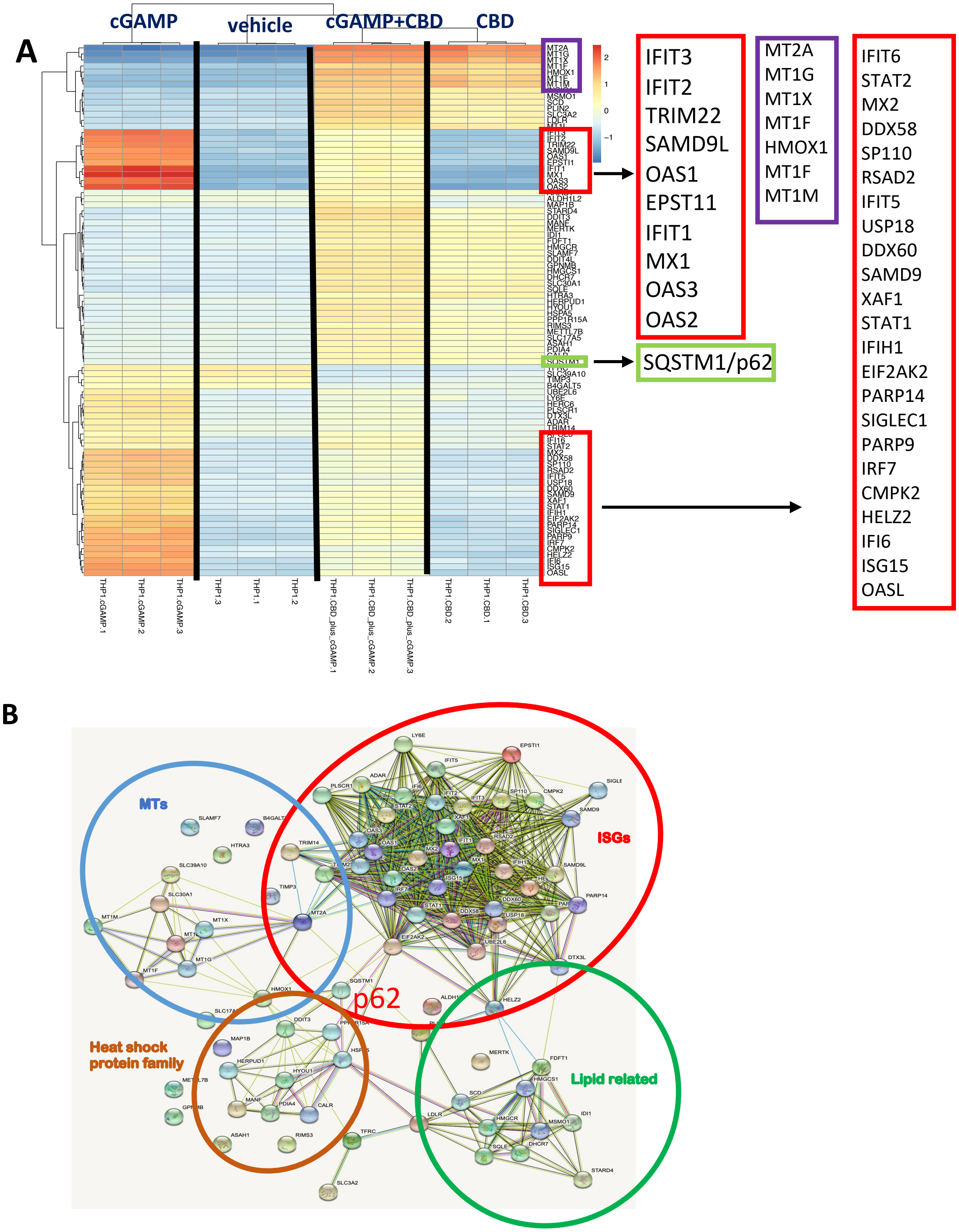

Supplementary Figure 1. Differential gene expression and functional protein association network (STRING) analysis of RNA-seq data. THP-1 cells were treated with vehicle or 10ug/ml CBD and co-stimulated with 2’3’-cGAMP for 8 hours. Afterwards, we extracted RNA for transcriptomics analysis through RNA-seq. (A) Differential gene expression of THP-1 that were mock treated, or treated with 2’3’-cGAMP alone, CBD alone, or cotreated with CBD and 2’3’-cGAMP. Gene families belonging to metal homeostasis is highlighted in purple box; ISGs are highlighted in red boxes; autophagy receptor SQSTM1/p62 is highlighted in green box. (B) Protein-protein interactions and pathway analysis by protein association network (STRING) analysis.

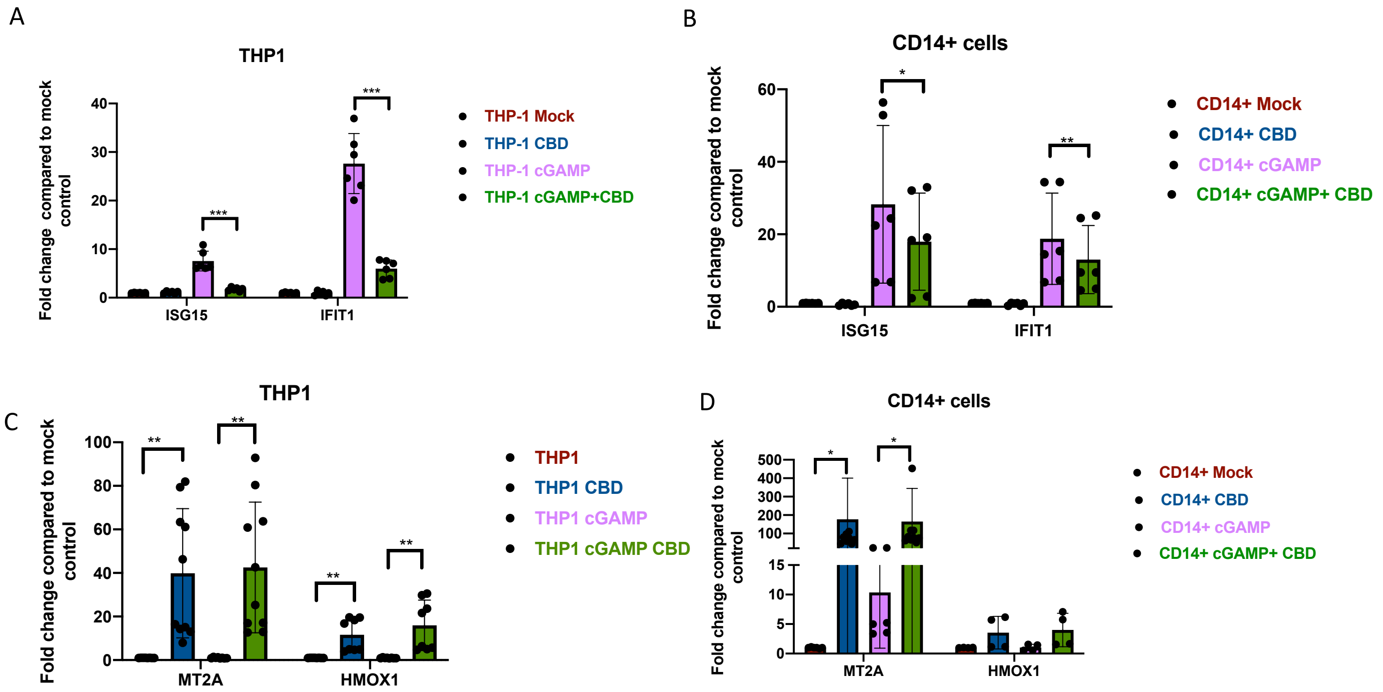

Supplementary Figure 2. Confirmation of RNA seq results by RT-PCR. THP-1 and primary CD14+ macrophages were treated with either mock, CBD alone, 2’3’-cGAMP or 2’3’-cGAMP and CBD for 8 hours. CBD attenuate 2’3’-cGAMP upregulation ISG15 and IFIT1 in (A) THP-1 and, (B) and primary macrophages. CBD upregulate MT2A and HMOX1 regardless of 2’3’-cGAMP stimulation in (C) THP-1 and, (D) primary macrophages.

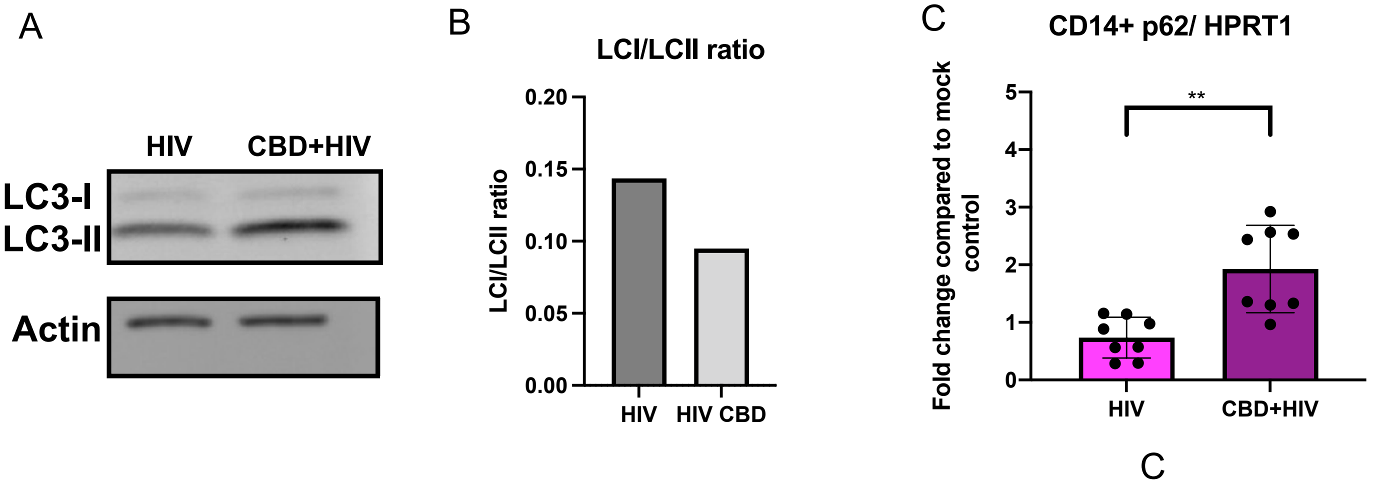

Supplementary Figure 3. Increased autophagy activity in CBD treated HIV infected primary macrophages. CD14+ primary monocytes sorted from healthy PBMCs were first differentiated into macrophages then treated with vehicle or 2ug/ml CBD for 1 day, followed by HIV infection for 8 days in the absence or presence of 2ug/ml CBD. (A) Western blot of HIV and HIV+CBD treated cells (B) Bar graph showing LCI/LCII ratio of the western blot analyzed by Image Lab Software. (C) Real time PCR analysis of P62 mRNA expression in HIV infected cells with or without CBD treatment.
